# Turkish Chronic Myeloid Leukemia Study: Retrospective Sectional Analysis of CML Patients

**DOI:** 10.4274/tjh.2013.0151

**Published:** 2013-12-05

**Authors:** Fahri Şahin, Güray Saydam, Melda Cömert, Burak Uz, Akif Selim Yavuz, Esra Turan, İpek Yönal, Hilmi Atay, Engin Keltikli, Mehmet Turgut, Mustafa Pehlivan, Meltem Olga Akay, Emel Gürkan, Semra Paydaş, Selda Kahraman, Fatih Demirkan, Onur Kırkızlar, Seval Akpınar, Gülsüm Emel Pamuk, Muzaffer Demir, Hasan Mücahit Özbaş, Mehmet Sönmez, Mine Gültürk, Ayşe Salihoğlu, Ahmet Emre Eşkazan, Cem Ar, Handan Haydaroğlu Şahin, Şeniz Öngören, Zafer Başlar, Yıldız Aydın, Mustafa Nuri Yenere, Nükhet Tüzüner, Burhan Ferhanoğlu, İbrahim C. Haznedaroglu, Osman İlhan, Teoman Soysal

**Affiliations:** 1 Ege University Faculty of Medicine, Department of Hematology, İzmir, Turkey; 2 Hacettepe University Faculty of Medicine, Department of Hematology, Ankara, Turkey; 3 İstanbul University Faculty of Medicine, Department of Hematology, İstanbul, Turkey; 4 Ondokuz Mayıs University Faculty of Medicine, Department of Hematology, Samsun, Turkey; 5 Gaziantep University Faculty of Medicine, Department of Hematology, Gaziantep, Turkey; 6 Osmangazi University Faculty of Medicine, Department of Hematology, Eskişehir, Turkey; 7 Çukurova University Faculty of Medicine Department of Hematology and Oncology, Adana, Turkey; 8 Dokuz Eylül University Faculty of Medicine, Department of Hematology, İzmir, Turkey; 9 Trakya University Faculty of Medicine, Department of Hematology, Edirne, Turkey; 10 Karadeniz Technical University Faculty of Medicine, Department of Hematology, Trabzon, Turkey; 11 İstanbul University Cerrahpaşa Faculty of Medicine, Department of Hematology, İstanbul, Turkey; 12 Ankara University Faculty of Medicine, Department of Hematology, Ankara, Turkey

**Keywords:** Chronic myeloid leukemia, Registry, Response

## Abstract

**Objective:** here have been tremendous changes in treatment and follow-up of patients with chronic myeloid leukemia (CML) in the last decade. Especially, regular publication and updating of NCCN and ELN guidelines have provided enermous rationale and base for close monitorization of patients with CML. But, it is stil needed to have registry results retrospectively to evaluate daily CML practices.

**Materials and Methods:** In this article, we have evaluated 1133 patients’ results with CML in terms of demographical features, disease status, response, resistance and use of second-generation TKIs.

**Results:** The response rate has been found relatively high in comparison with previously published articles, and we detected that there was a lack of appropriate and adequate molecular response assessment.

**Conclusion:** We concluded that we need to improve registry systems and increase the availability of molecular response assessment to provide high-quality patient care.

**Conflict of interest:**None declared.

## Abstract (tr)

**Amaç:** Kronik myeloid lösemi (KML) tanısı olan hastalarda tedavi ve takip kriterleri son 10 yılda çok ciddi değişimlere uğramıştır. Özellikle NCCN ve ELN kılavuzlarının yayınlanması ve düzenli olarak güncellenmesiyle hastaların daha yakın takibi mümkün olmuştur. Ancak güncel uygulamaları değerlendirebilmek için kayıt ve retrospektif analizlere ihtiyaç vardır.

**Gereç ve Yöntemler:** Bu çalışmada, ülkemizde 12 merkezin katılımıyla KML tanısıyla izlenen 1133 hastanın demografik özellikleri, hastalık durumları, yanıt, direnç ve ikinci kuşak tirozin kinaz inhibitör kullanımları analiz edilmiştir.

**Bulgular:** Hasta grubumuzda, yanıt oranlarının litaretüre göre daha yüksek olduğu, moleküler yanıt değerlendirmenin tüm merkezlerde mümkün olmadığı saptanmıştır.

**Sonuç:** KML hastalarının bakım kalitesinin arttırılması için moleküler teknik kullanımının arttırılması ve daha yaygın kayıt sistemine ihtiyaç vardır.

## INTRODUCTION

Introduction Chronic myeloid leukemia (CML) is a clonal myeloproliferative disease characterized by t(9:22) translocation, which produces the BCR-ABL fusion gene [1]. It is very well documented that the expression of constitutively activated tyrosine kinase, which is a product of BCR-ABL, is the underlying reason for the CML phenotype [2,3]. The reciprocal translocation between chromosomes 9 and 22 produces the shortened 22q known as the Philadelphia chromosome (Ph), and the new fusion gene is called the BCR-ABL fusion gene [4]. The clinical presentation of CML in most cases is seen in 3 different clinical phases: the chronic phase, accelerated phase, and blastic phase [5]. If left untreated, newly diagnosed chronic-phase CML patients finally progress to the accelerated and blastic phases. The blastic phase is of mainly the myeloid phenotype in almost two-third of patients and of the lymphoid phenotype in most of the remaining patients [6]. The blastic phase of the disease in all forms has a poor prognosis, with overall survival of 3 to 6 months. Most of the patients have been diagnosed in chronic phase [7]. CML has been treated with hydroxyurea, interferon, chemotherapy, and, most effectively, allogeneic stem cell transplantation. After 2000, the treatment strategy and results were completely changed by the introduction of targeted treatment with tyrosine kinase inhibitor (TKI) imatinib. The tyrosine kinase activity of BCR-ABL is the main therapeutic target of imatinib, the first TKI to be used in the treatment of CML. A large phase III randomized trial, known as IRIS, provided the clinical and scientific background for the use of imatinib in the treatment of Ph (+) leukemias [8]. At a median follow-up of 19 months, the rate of complete cytogenetic response (CCyR) in the imatinib-treated patients was 94%, compared with a CCyR of 8.5% achieved by patients in the IFN-alpha and cytarabine arm. According to the 5-year results of the IRIS study, only 68% of the patients in CCyR still remained on imatinib therapy [9]. The previously reported prospective IRIS trial, which retrospectively compared patient groups treated with imatinib and interferon, and single-center studies and comparison of allogeneic stem cell transplantation with data reported from stem cell transplant registries have confirmed the superiority of imatinib treatment to previously used strategies [9,10,11]. However, there are only a few reports describing imatinib therapy in patients with CML treated outside prospective trials or even from CML patient registries [12,13]. Although there have been tremendous increases in knowledge regarding clinical and molecular features of CML, epidemiology and treatment of CML in daily practice have not been studied in detail [14]. Sources of epidemiological data are mainly mortality statistics, European cancer registries such as the Swedish Cancer Registry or the Saarland Registry in Germany, or the database of the Surveillance, Epidemiology, and End Results Program of the United States National Cancer Institute [15]. 

The European LeukemiaNet (ELN) has developed recommendations for the medical management of patients with CML in daily clinical practice [15]. A careful and close monitoring of treatment response and of prognostic factors is required first to identify development of first-line therapy (imatinib) resistance, intolerance, and noncompliance or progression to advanced-phase disease. Subsequently, the treatment benefits of second-line therapies have to be considered. For success of treatment strategies, all required data and monitoring schedules of patients should be recorded appropriately. 

There have not been many studies of treatment, follow-up, and monitoring strategies of patients with CML treated with TKIs in Turkey. Recently, Saydam et al. published the results of patients with CML treated with dasatinib under a compassionate use program [7]. However, that article covered only a small segment of patients with CML, was not nationwide in scope, and did not focus on the general CML population. 

The aim of this report was to determine the demographic features, disease characteristics, treatment and monitoring strategies, response status, and survival rates of patients with CML treated with TKIs in Turkey. 

## MATERIALS AND METHODS

**Data Collection**

This study was designed as a retrospective sectional study. The primary objective of this study was to evaluate the patients with the diagnosis of CML in Turkey. To collect the essential and maximum available data on patients with CML, a steering committee was organized and met to define the required information and to create a standardized questionnaire. The questionnaire consisted of separate sections such as demographical data and patient characteristics, disease characteristics, therapy and side effects, and, finally, last status of the patients. Across all of Turkey, 11 centers were enrolled in the study and 2 physicians from each center were chosen to fill out the forms. Ethics committee approval was obtained from the Ege University Ethics Committee with the date of 22 November 2011 and number of B.30.2.EGE.0.20.05.00/BOY/1401/575. The first patient was recorded on 13 August 2012. The data collection process was finished by October 2012 and statistical analyses were completed by the end of January 2013. 

**Patients’ Inclusion**

Patients of ≥18 years old with the diagnosis of CML irrespective of the diagnosis date and treatment strategies were enrolled in the study if the required data could be provided by the primary physicians. 

**Patients’ Exclusion**

Patients were excluded if they did not have cytogenetically and/or molecularly confirmed diagnosis of CML at any time point in their follow-up. Patients who could not have regular follow-up or had interruption in their follow-up of longer than 1 year, those who were referred for allogeneic stem cell transplantation, and those who stopped CML treatments were also excluded. 

**Treatment and Monitoring**

All patients with the diagnosis of CML were included irrespective of their current treatment, with the exception of allogeneic stem cell transplantation. The duration of the current treatment, dosage, dose and therapy changes, and side effects were recorded. If there was more than one treatment in the patient’s history, the same information regarding these other therapies was also obtained. Information on monitoring was classified as the duration, time points, and results based on the ELN recommendations for hematological, cytogenetic, and molecular responses. The results for each time point were not evaluated independently and separately; instead, certain definitions such as complete hematological response (CHR), minor/minimal/partial/major cytogenetic response (CyR), and major molecular response (MMR) or complete molecular response (CMR; undetectable BCR-ABL transcripts with currently available techniques) were used as recommended by and indicated in the National Comprehensive Cancer Network (NCCN) and ELN guidelines. 

**Endpoints**

The primary endpoint of this program was to provide information regarding clinical, demographic, laboratory, and treatment status of patients with the diagnosis of CML, and also to determine the response rates, number of treatment-related adverse events, and use of second-generation TKIs. Dose modifications, disease status under TKI treatment, and cytogenetic and molecular responses were also analyzed and evaluated according to the available patient data. 

**Statistical Analysis**

All the statistical analyses were performed by using the data obtained from the patients’ files as recorded by primary physicians. A special form was designed to summarize the required data; it was completed by primary physicians and analyzed by an independent contract research organization. Any patient who received the diagnosis of CML at any time point was included in the evaluation. Demographics, disease status at baseline, time from diagnosis, duration of treatment, the reasons for switching therapy to dasatinib and/or nilotinib, and the median dose of imatinib were summarized for all patients. Additionally, the last disease status, mortality rates, discontinuation rates, use of second-generation TKIs, and reported adverse events and dose modifications were also presented. Statistical analyses were performed by using SPSS 20 and Excel 2007. The variables were first assessed by Kolmogorov–Smirnov/Shapiro–Wilk testing in terms of normal distribution. The results were provided as mean±SD for normally distributed variables and as median (min-max) for abnormally distributed parameters. Time to progression (TTP) was defined as the time between starting the drug and either discontinuation/switching of the drug for any reason or death. Overall survival (OS) was defined as the time period between the time of diagnosis to death because of any reason as well as any death reported after the drug was stopped. TTP and OS evaluations were performed by using the Kaplan–Meier method. 

## RESULTS

The demographic features of patients at the time of diagnosis are illustrated in Table 1. Based on these data, median age was calculated as 46.1±14.8 years for all patients, and this was similar for both sexes in terms of time of diagnosis. There was no difference between the rates of male and female patients, as 50.7% of patients were female and 49.3% were male. It was noticed that all patients had splenomegaly within the range of a median of 5 cm up to 40 cm. Hepatomegaly was detected in 46.5% of patients. 

Disease status of patients at the time of diagnosis is illustrated in Table 2. The median white blood cell (WBC) count was calculated as 101x10^3^/mm^3^ (range: 29x102 to 14x106). The median eosinophil percentage was 2.5% (range: 0%-9%), while the basophil level was 3±5%. Median hemoglobin level at the time of diagnosis was 11.5 g/dL, while platelet count was 275.10^3^/mm^3^ (range: 22.104 to 24.105/mm^3^). At the time of diagnosis, 77.5% of patients had bone marrow fibrosis of any degree, and 83.2% of patients had hypercellular bone marrow histology. During first evaluation of patients during diagnostic work-up, 94.9% of patients were in the chronic phase, 4.1% were in the accelerated phase, and 1.1% were in the blastic phase. Sokal risk scores at diagnosis were calculated as low in 575/831 (69.2%), intermediate in 201/ 831 (24.2%), and high in 55/831 (6.6%) among patients with available data. Most of the patients (76.4%) had been treated with hydroxyurea after diagnosis in terms of decreasing WBC count before starting imatinib. The median dose of imatinib was reported as 400 mg/day (range: 100-800 mg) and median duration of imatinib therapy was 35.6 (range: 0.7-275.5) months. Since this evaluation does not have limitations in terms of therapy and diagnosis duration and it includes all patients with the diagnosis of CML, treatment duration with imatinib has a large range at 0.7 months to 275.5 months. 

All patients had imatinib as the first line of therapy regardless of the phase of the disease. The most prominent reported side effects of imatinib were as follows: cytopenias in more than one lineage (10.75%), edema (6.41%), thrombocytopenia (4.67%), nausea (3.91%), rashes in grades 1-2 (3.04%), musculoskeletal pain (2.61%), neutropenia (2.39%), leukopenia (1.85%), vomiting (1.52%), malaise (1.41%), and “others”, which covers mainly local edema, increase in biochemical parameters, and gastrointestinal disturbance (13.24%). Most of these side effects were managed successfully Table 3. 

Response to treatment with imatinib was evaluated in terms of hematological, cytogenetic, and molecular response based on current and previously published NCCN and ELN guidelines. This evaluation was performed by patients’ primary physicians and the available data collected from screening forms were statistically analyzed. Based on this analysis, 95.7% of patients treated with imatinib had CHR and 63.8% of patients had CCyR at certain time points. Molecular response evaluation could not be performed due to lack of available data in most of the patients. Response to imatinib therapy and results are provided in Table 4. When we checked the progression to accelerated and blastic phases under imatinib treatment, it was noted that 114 of 1133 patients (10.1%) had progressed. The rates of progression in all Sokal risk score groups were similar at 10.1% in all groups. Median time to progression was 58.5±30.1 months. 

Table 5 summarizes the use of second-generation TKIs (dasatinib, nilotinib, or both) after imatinib failure or intolerance. Imatinib had to be replaced by dasatinib or nilotinib, or sequentially by both, in 332 patients (29.3% of total) and, of those, 307 (90.8%) had to have their imatinib therapy changed due to resistance/inadequate response and 25 (9.2%) had to have it changed because of dug intolerance. The first choice for switching TKI therapy in 194 patients (58.8%) was dasatinib, in 138 patients (41.2%) it was nilotinib, and 114 patients had to use both drugs in the course of CML due to either failure or intolerance. When the response to second-generation TKIs was evaluated, CCyR was calculated as 31.3% in patients treated with nilotinib and/or dasatinib. Due to lack of available data, molecular response could not be assessed. 

**Figure 1:**
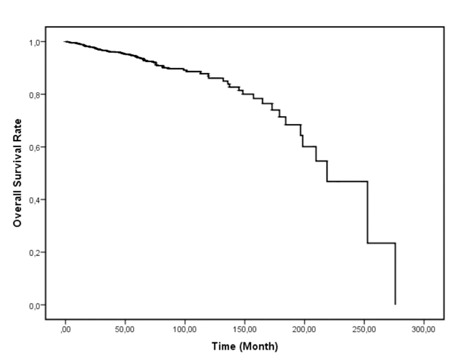
Overall survival analyses for all patients with the diagnosis of CML irrespective of treatment.

At the end of study and data collection period, 86 (7.6%) patients were deceased and 1047 (92.4%) patients were alive (Figure 1). The median survival time for all patients was 218 (0.7-245.6) months. 

**Figure 2:**
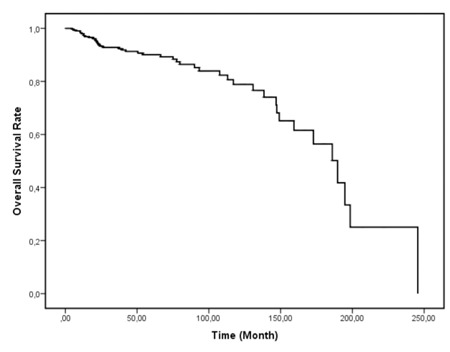
Overall survival plot after switching therapy to second-generation TKIs.

The OS time was recalculated after switching imatinib therapy to nilotinib or dasatinib and Kaplan–Meier survival estimation resulted in 189.7 (0-275.9) months of OS for those patients (Figure 2). 

## DISCUSSION

In this study, we have evaluated 1133 Turkish patients with the diagnosis of CML in terms of demographic characteristics and disease status, treatment strategies and switching rates, and side effects. We have found that, during the first evaluation of patients during diagnostic work-up, 94.9% of patients were in the chronic phase, 4.1% were in the accelerated phase, and 1.1% were in the blastic phase. All patients had imatinib as a first-line therapy regardless of the phase of the disease. It was found that 95.7% of patients treated with imatinib had CHR and 63.8% of patients had CCyR at certain time points. Molecular response evaluation could not be performed due to lack of available data in most of the cases. When we checked the progression to accelerated and blastic phases under imatinib treatment, it was noted that 114 of 1133 patients (10.1%) had progressed. Median time to progression was 58.5±30.1 months. Imatinib has to be replaced by dasatinib or nilotinib, or sequentially by both, in 332 patients (29.3% of total) and, of those, 307 (90.8%) had to change their imatinib therapy due to resistance/inadequate response and 25 (9.2%) had to change imatinib therapy because of drug intolerance. The first choice for switching TKI therapy in 194 patients (58.8%) was dasatinib, in 138 patients (41.2%) it was nilotinib, and 114 patients had to use both drugs in the course of CML due to either failure or intolerance. 

Our study has been the first nationwide CML registration study with the largest enrolled patient population. Since it is not a prospective trial and does not have any time limitations, it may be accepted as a reflection of current CML practice in Turkey outside of clinical trials. Patients participating in clinical trials are usually selected according to strict eligibility criteria. However, in practical situations, the clinical features of patients are much more heterogeneous than those defined by the selection criteria in clinical trials. Because of that, sometimes, the results of clinical trials might not be applicable to real medical practice. However, it is very obvious that the results of treatment with TKIs outside of clinical trials are mandatory in order to evaluate and prove the efficacy of TKIs and for confirmation of clinical trials. 

The comparison of results obtained from clinical trials with results of patients in routine practice has always been controversial. To do this successfully, dedicated registry programs are required with well-defined parameters. TARGET (Timely and Appropriate Registration System for GLIVEC Therapy) is a Japanese organization to improve the quality of medical care for CML patients in Japan [16]. The TARGET system is an online database that can be easily accessed by physicians. Results of patients registered in the TARGET system from 2003 to 2010 were recently published [17]. In that study, Tauchi et al. evaluated 639 CML patients followed for 90 months and treated with imatinib as a first-line therapy. They reported high survival rates with event-free survival (EFS), progression-free survival (PFS), and OS at 79.1%, 94.8%, and 95.1%, respectively. Of course, it is not possible to compare these results with ours, as their patient population was highly homogeneous and was followed more strictly compared to ours. 

There are other registry studies reporting the results of CML patients in terms of changing trends and survival plots. Björkholm et al. published the Swedish registry results of 3173 diagnosed patients who were followed for the last 36 years [18]. They reported that the survival rates of patients changed dramatically after introduction of imatinib into clinical practice and that the estimated survival of patients with CML could be prolonged by up to 79 years by appropriate use of imatinib. These data, however, were sorted from the Swedish Cancer Registry and are not specific for only CML; they also lack available data on PFS and EFS [18]. All the same, the study was very important because of its large number of patients and because it provided relative survival rates in terms of changing paradigms of CML treatments. 

CAMELIA is an international population-based, non-interventional, observational multicenter clinical registry system established by the Czech and Slovak Society of Hematology in 2004 [19]. Recently, they published 661 consecutive CML patients registered to this system in terms of the use of imatinib in first- or second-line treatment and the role of stem cell transplantation in this patient cohort. However, these patients were entered into the system between the years of 2000 and 2008, and some of them could have begun imatinib therapy relatively late. They proposed that, for success of not only imatinib therapy but also of stem cell transplantation, timing and appropriate dosing have been important factors influencing the results. Some of our patients, as in the case of CAMELIA, were diagnosed before the introduction of imatinib, but they began being treated with imatinib immediately after its approval. We also did not aim to investigate the role of stem cell transplantation in our patient cohort. 

Our study, unfortunately, could not rely on any registry system, and the parameters for analyses were designed by other investigators before data were collected. Since this was not a prospective study, but rather was performed based on retrospective patients’ files, the lack of some data was seen due to inadequate records. However, one must not forget that this study includes the largest patient cohort ever assembled in Turkey to date, and it is a good reflection of the current status of CML therapy and results currently available. We have the hematological and cytogenetic results of almost all patients, but unfortunately we do not have much information about molecular responses. Molecular response to TKI therapy in patients with CML could not be assessed in our retrospective study due to lack of available and standardized real-time RT-PCR results, the universally accepted technique for detection of BCR-ABL transcripts. Although a small part of the participating centers in this sectional study have been involved in ELN standardization projects, also known as the EUTOS Project, most of the centers do not have laboratory facilities for obtaining standardized PCR results [20]. Recently, major hematology clinics have started to use commercially available kits with international scale values. 

Our study has clarified that appropriate and adequate recording systems and, furthermore, dedicated and specific, non-interventional, and prospective recording systems are mandatory for not only future projects and research but also for patient care and effective follow-up. Our study also confirmed that all Turkish CML patients have similar hematological and cytogenetic response results as those reported by clinical trials and national registry programs. However, it is obvious that, for molecular evaluation, much more effort is required in terms of establishment of adequate PCR facilities, which should be standardized eventually. 

**Acknowledgments**

We would like to thank all colleagues who provided the data of their patients for this analysis. 

## CONFLICT OF INTEREST STATEMENT

The authors of this paper have no conflicts of interest, including specific financial interests, relationships, and/ or affiliations relevant to the subject matter or materials included. 

## Figures and Tables

**Table 1:**
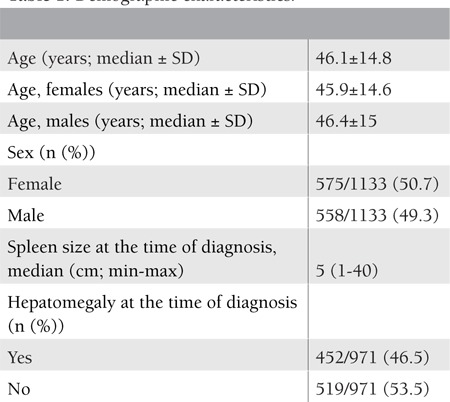
Demographic characteristics.

**Table 2:**
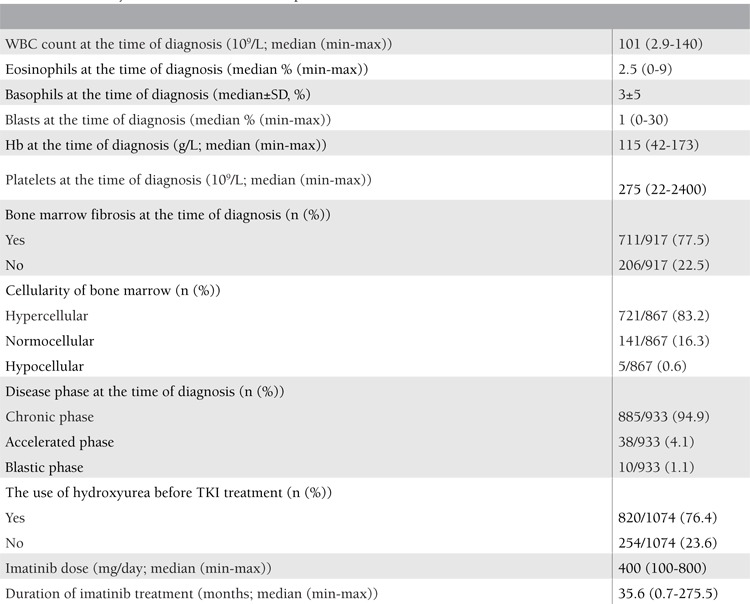
Laboratory characteristics and disease phases

**Table 3:**
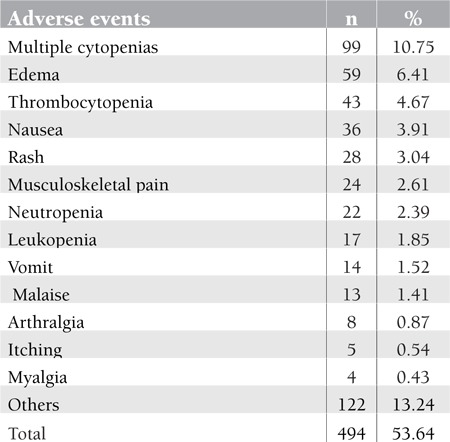
The frequency of adverse events during imatinibtreatment.

**Table 4:**
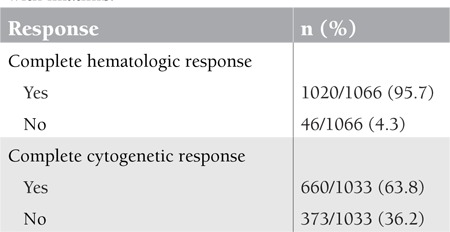
Response evaluation in CML patients treated with imatinib

**Table 5:**
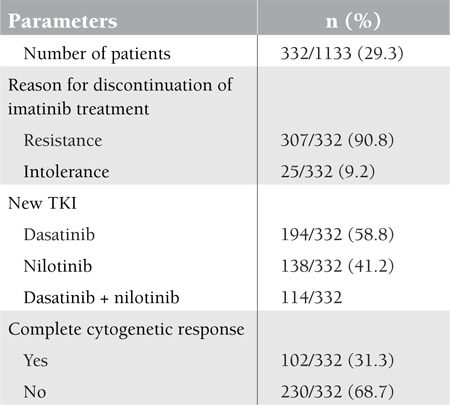
The general characteristics of second-line treatment after imatinib failure/intolerance
